# Urine Extracellular Vesicles Size Subsets as Lupus Nephritis Biomarkers

**DOI:** 10.3390/diagnostics14202271

**Published:** 2024-10-12

**Authors:** Itze C. Navarro-Hernandez, Raúl F. Reyes-Huerta, Mariana Cañez-Hernández, Jiram Torres-Ruiz, Daniel A. Carrillo-Vázquez, Laura P. Whittall-García, David E. Meza-Sánchez, Guillermo Juárez-Vega, Diana Gómez-Martin, José M. Hernández-Hernández, José L. Maravillas-Montero

**Affiliations:** 1 B Cell Immunology Laboratory, Coordinación de la Investigación Científica, Universidad Nacional Autónoma de México, Mexico City 04510, Mexico; itze.navarro9@gmail.com (I.C.N.-H.); dmeza@cic.unam.mx (D.E.M.-S.); 2 Departmento de Biología Celular, Centro de Investigación y de Estudios Avanzados del Instituto Politécnico Nacional, Mexico City 07360, Mexico; 3 Doctorado en Ciencias Biomédicas, Universidad Nacional Autónoma de México, Mexico City 04510, Mexico; 4 Departamento de Inmunología y Reumatología, Instituto Nacional de Ciencias Médicas y Nutrición Salvador Zubirán, Mexico City 14080, Mexico; 5 Red de Apoyo a la Investigación, Instituto Nacional de Ciencias Médicas y Nutrición Salvador Zubirán y Universidad Nacional Autónoma de México, Mexico City 04510, Mexico; guillermovega@cic.unam.mx

**Keywords:** extracellular vesicles, lupus nephritis, urine, biomarker

## Abstract

Systemic lupus erythematosus (SLE) is an autoimmune disorder that often leads to kidney injury, known as lupus nephritis (LN). Although renal biopsy is the primary way to diagnose LN, it is invasive and not practical for regular monitoring. As an alternative, several groups have proposed urinary extracellular vesicles (uEVs) as potential biomarkers for LN, as recent studies have shown their significance in reflecting kidney-related diseases. As a result, we developed a flow cytometry approach that allowed us to determine that LN patients exhibited a significantly higher total uEV concentration compared to SLE patients without kidney involvement. Additionally, an analysis of different-sized uEV subsets revealed that microvesicles ranging from 0.3 to 0.5 μm showed the most promise for distinguishing LN. These findings indicate that evaluating uEV concentration and size distribution could be a valuable diagnostic and monitoring tool for LN, pending further validation in more comprehensive studies.

## 1. Introduction

Extracellular vesicles (EVs) are membrane-contained structures secreted by most cell types and are considered central messengers for intercellular communication [1]. These EVs typically contain definite proteins, lipids, and nucleic acids such as microRNAs, tRNA-derived small RNAs, and long non-coding RNAs [2]. They can be categorized into three main classes depending on their cellular source and relative size: exosomes (30–100 nm), microvesicles (100 nm–1 μm), and apoptotic bodies (1–5 μm) [3]. EVs are typically obtained from bodily fluids, such as blood (plasma or serum), saliva, and urine. This accessibility allows for non-invasive sample collection [4,5]. Moreover, EVs represent highly stable particles contained in many samples, besides their molecule enrichment degree or specific molecule presence, which can correlate with the status of many health disorders [6,7,8,9,10].

Urinary EVs (uEVs) present a unique advantage for studying renal diseases, as they can reflect significant pathological changes in the kidneys [11,12,13,14]. These vesicles are produced by different cell types within the kidney, such as podocytes, tubular epithelial cells, and endothelial cells. Their composition can vary depending on kidney physiological and pathological conditions [15,16]. 

Lupus nephritis (LN) is one of the most severe complications and a leading cause of death in Systemic Lupus Erythematosus (SLE). LN is a form of glomerulonephritis with deposition of immune complexes (ICs) and complement, which results in renal tissue damage in the kidneys of LN patients, endothelial damage, and microthrombi formation [17]. Although renal biopsy has long been considered the “gold standard” for diagnosis of LN [18], its invasive nature and limited scope of renal tissue analysis warrant a more advanced approach. The need for non-invasive and sensitive diagnostic and prognostic methods is evident. Hence, discovering new biomarkers would improve diagnosis and offer additional insights into disease activity, treatment selection, and patient follow-up [19,20]. 

Interestingly, it has been reported that EVs play a role in developing LN through various mechanisms. These mechanisms include EVs as a source of extracellular or intracellular autoantigens and complement activators [21]. Beyond their pathogenic implications, these uEVs could be proposed as a “liquid biopsy” approach alternative in LN beyond or with conventional blood-associated variables. Recent works have reported their utility in identifying or following up on LN patients by analyzing specific content of the uEVs [22] but ignoring their intrinsic properties, such as their size distribution or abundance. Thus, we decided to study these urinary EVs’ features that could represent potential biomarkers for LN patients, employing a simple flow cytometry approach.

## 2. Methods

### 2.1. Sample Collection

First morning urines (15 mL) were collected from 15 LN patients and 11 SLE patients with no kidney involvement at the time of sampling. All the samples were stored at −20 °C until used. Laboratory and clinical features confirmed SLE patients without kidney involvement. Besides using the same approach to assess a diagnostic, the patients with LN were assessed by a percutaneous renal biopsy and then classified by their displayed glomerular disease type by means of the criteria established by the International Society of Nephrology/Renal Pathology Society (ISN/RPS) [23]. Remarkably, all urine samples were obtained from patients no longer than three weeks after performing the renal biopsy procedure. Exclusion criteria included pregnancy or puerperium, any ongoing acute or chronic infection, neoplasia, or patients treated with any biological therapy. All patients in both groups were under immunosuppressive treatment at the time of recruitment. The majority were under prednisone treatment (LN 81 vs. non-LN 90%) and hydroxychloroquine (LN 73.1 vs. non-LN 80%) in both groups. The other most frequent treatments in the lupus nephritis group were cyclophosphamide (LN 35 vs. non-LN 0%) and mycophenolate mofetil (LN 23 vs. non-LN 0%), and in the non-lupus nephritis group were azathioprine (non-LN 25 vs. LN 12%) and methotrexate (non-LN 30 vs. LN 3.8%). None were under B cell-depleting drugs. The characteristics of all these individuals are depicted in Table 1.

All recruited patients signed informed consent forms before being included in our study. The Institutional Ethics Committee of the Instituto Nacional de Ciencias Médicas y Nutrición Salvador Zubirán reviewed and approved our protocol (Ref. 2555) in compliance with the Helsinki Declaration.

### 2.2. Extracellular Vesicles Isolation

The urinary extracellular vesicles were isolated, as we previously described, with a protocol validated to minimize the loss and deterioration of EVs [24]. In brief, whole urine (15 mL) was centrifuged at 3000× *g* for 10 min at 4 °C to remove all cell debris and urinary cells. Subsequently, the supernatant was collected and centrifuged at 10,000× *g* for 45 min at 4 °C to dispose of considerable molecular weight particle contamination. The pellet was treated with β-mercaptoethanol to remove the interfering Tamm–Horsfall (THP) protein to obtain a good yield of uEVs. The residual supernatant was then centrifuged at 160,000× *g* for 70 min at 4 °C. Finally, the supernatant was discarded, and the pellet containing uEVs was washed and resuspended in 1 mL of 1X PBS buffer with protease inhibitors (uEVs “stock”). Isolated uEVs were stored at −70 °C until use.

### 2.3. Flow Cytometry

A total of 20 µL of each uEVs prepared stock was stained with 0.4 µL of carboxyfluorescein succinimidyl ester (CFSE, Thermo Fisher Scientific, Waltham, MA, USA) [5 nM] for 10 min at 37 °C to allow discrimination between the “background noise” (debris) in the flow cytometry analysis. Samples were then diluted with 400 µL of cold sterile PBS 1x for immediate analysis on an Accuri C6 cytometer (Becton Dickinson, Franklin Lakes, NJ, USA). The usage of this type of cytometer is highly relevant since it is equipped with a fixed flow rate with peristaltic pumps that allow fine-tuning and control of sample volumes. Cytometer capture parameters were set at 100 μL of total volume at a slow flow rate. Defined-size gating was achieved employing Megamix Plus-FSC (BioCytex, Marseille, France) fluorescent (FL1) beads with the following diameters: 0.1 µm, 0.3 µm, 0.5 µm and 0.9 µm. This mix was used to establish the area of interest covering each most approximate EV size. This method is also detailed by Navarro-Hernandez et al. [24]. Finally, all data were processed using FlowJo v10.10 software (Becton Dickinson, Franklin Lakes, NJ, USA), and the acquired results were normalized using the creatinine in the urine values of each sample to obtain the relative uEV concentration (total or defined by particle size).

### 2.4. Statistical Analysis

Differences in uEVs between groups were assessed with a Mann–Whitney U test. Additionally, we generated a correlation matrix using Spearman’s rank correlation test between uEV concentrations and the clinical features of SLE/LN patients. Receiver operating characteristic (ROC) curves were generated to calculate the area under the curve (AUC) for discernment between LN or non-LN groups. All performed analyses were accomplished using the Prism (GraphPad) v. 10.3.0 software and the R platform, v. 4.0.2 (R Foundation for Statistical Computing, Vienna, Austria; available online: http://www.R-project.org/ (accessed on 4 July 2024).

## 3. Results

### 3.1. Urinary EV Concentrations Are Elevated in LN

**Figure 1:**
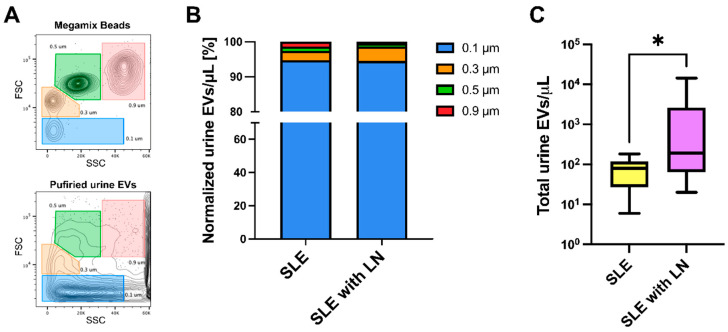
uEVs display no differences in size distribution but exhibit increased concentration in LN. (**A**) Gates for each bead size (upper panel) employed as a reference to define the relative size of purified uEVs in patient samples (lower panel, showing a representative contour plot). FSC—forward scatter (size); SSC—side scatter (granularity). (**B**) Size distribution plot displaying normalized mean frequencies of each uEV size subset in SLE vs. LN patients. (**C**) Total uEV concentration in SLE vs. LN patients. The data were analyzed using the Mann–Whitney U test. * *p* < 0.05.

Employing a previously standardized method that allows setting our flow cytometer to study uEVs within delimited size gates and obtaining reproducible counts, we defined four bead size-defined plot regions to identify particles from 0.1 to 0.9 μm (Figure 1A). Then, we analyzed the enriched uEV preparations from patients’ samples using these parameters, excluding most of the associated debris. Once normalized, uEV counts were compared between lupus and LN-exhibiting patients, finding that 0.1 μm EVs were the most represented ones in both groups but exhibited no differences in the percentual size distribution of urinary vesicles regarding any subset analyzed (Figure 1B). However, we found that the total (all evaluated sizes) uEV concentration was significantly higher in LN patients (Figure 1C). When we analyzed each size-subset concentration, we also found that each of the 0.1, 0.3, and 0.5 μm EVs were significantly more abundant in LN patients’ urine (Table 2).

### 3.2. uEV Concentrations Correlate with Clinical and Laboratory Variables in SLE and LN

**Figure 2:**
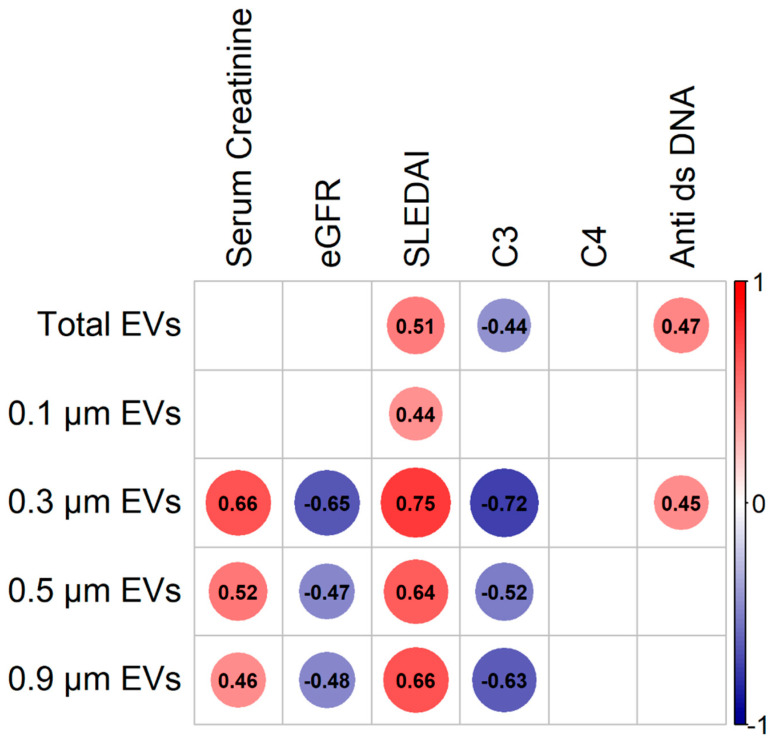
uEV levels correlate with different clinical features in SLE/LN patients. Correlation matrix of the calculated Spearman coefficient between uEV concentrations of different sizes (left side) and clinical characteristics of SLE/LN patients (upper side). The sidebar color scale (right side) indicates the magnitude of a positive correlation (red) or a negative correlation (blue). The size of each circle represents the correlation coefficient enclosed. All the displayed correlations in the plot are significant (*p* < 0.05), besides the non-significant correlations that are represented with empty white squares.

To evaluate the clinical relevance of determining the uEV concentrations, we performed analyses of the correlation between the amounts of uEVs and clinical and laboratory features typically assessed in lupus in non-LN/LN patients (Figure 2). Remarkably, all uEV size-subset concentrations displayed significant positive and strong correlations with the Systemic Lupus Erythematosus Disease Activity Index (SLEDAI), and most of them also exhibited strong but negative correlations with serum concentration of the complement C3 subunit. Additionally, only the concentrations of total uEVs and 0.3 μm subset displayed moderated correlations with the serum amounts of antibodies’ anti-double stranded DNA (anti-dsDNA).

Interestingly, as shown in Figure 2, only the larger uEV subsets of 0.3, 0.5, and 0.9 displayed significant moderate to strong correlations with classical parameters used to evaluate renal status, such as serum creatinine levels (positive correlation) or the estimated glomerular filtration rate (eGFR, negative correlation).

### 3.3. uEVs as Urine Biomarkers of LN

**Figure 3:**
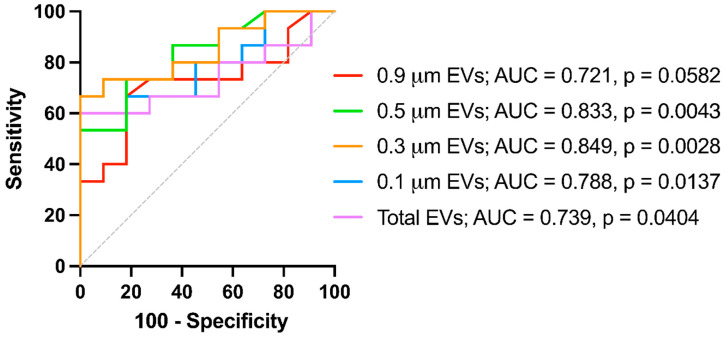
ROC curves of uEV concentrations for discrimination of LN patients and non-kidney affected SLE patients. The values of each AUC and *p*-values are depicted. *p*-values < 0.05 were considered significant. SLE patients (*n* = 11) and LN (*n* = 15).

Finally, to evaluate the utility of uEV concentration measurements as potential LN biomarkers, we generated receiver operating characteristic (ROC) curves for each vesicle size subset to determine their discriminative capacity in SLE vs. LN patients (Figure 3). We calculated the corresponding areas under the curve (AUC), and we concluded that both uEVs of 0.3 and 0.5 μm displayed significant outstanding discriminative values (AUC of 0.849 and 0.833, respectively). Still, even the 0.1 subset or total uEV concentration displayed significant, good values (AUCs above 0.7).

## 4. Discussion

Kidney damage leading to LN is one of the most common complications of SLE. In adult patients with SLE, as many as 5 out of 10 will develop kidney disease, increasing up to 8 out of 10 in children [25].

Percutaneous renal biopsy is considered the “gold standard” for diagnosing and classifying LN, besides its degree of activity or chronicity [26]. However, renal biopsy is a risky, invasive, and expensive surgical procedure that is not practical for monitoring LN due to the necessity of repetition [26]. Consequently, several serum and urinary biomarkers have been proposed to study SLE patients, focusing on LN. 

While numerous molecules have been suggested as LN diagnostic or prognostic biomarkers, such as metabolites, cytokines and chemokines, autoantibodies, and cell adhesion-related or micro-RNA molecules [27], the landscape of serum or uEVs has only recently started to receive attention [22]. However, these studies primarily obtained insight into the specific contents of these structures rather than the EVs themselves. This context led us to focus on the relative abundance and size distribution of these membranous entities, a novel and potentially impactful approach.

uEVs are secreted from urothelial cells, including those from the kidneys, ureters, bladder, urethra, and the male reproductive tract [28], with those derived from kidneys being the most abundant [29].

As expected, changes in uEV abundances have been marginally documented in the urinary tract or organ-associated related diseases, varying from no concentration differences among healthy subjects and prostate cancer patients [30] to an evident increase in acute kidney injury in cardiac surgery patients [31]. However, beyond the proposal of specific biomarker molecules associated with uEVs, such as different miRNAs or HMGB1 [21], there is a lack of information regarding the potential of determining the concentration or size distribution of uEVs in LN. Our study, which used a simple flow-cytometry-based method to stain and analyze EVs enriched from urine samples, found that the concentration of total or size-specific subsets of uEVs differs from LEG and LN-exhibiting patients. This result suggests the practical potential of uEVs as diagnostic or monitoring biomarkers for LN. 

First, we found that although no changes in size distribution were observed, the urine of individuals with LN exhibited a significant increase in total EV concentration. As mentioned before, there are only a few reports indicating concentration changes in uEVs during some chronic, deleterious kidney disorders, including their increased amounts in the urine of diabetic nephropathy [32]. Supporting the idea that urinary tract tissue damage induces these vesicles in urine, it has been reported that radiotherapy significantly increases uEV concentrations relative to pre-radiotherapy levels in patients with prostate cancer who develop bladder toxicity [33]. Interestingly, this study indicates that pretreatment urine samples displayed stable and normally distributed EV amounts, thus making plausible its usage to monitor biomarkers upon detection of aberrant vesicle concentrations. In this way, although our results are limited because of the cross-sectional type of the observations, the evident differences in the concentration of uEVs in LN patients and their correlation with different SLE or nephritis-associated variables point to a promising value of these structures as a diagnostic biomarker, with other potential applications that need to be evaluated by longitudinal approaches.

Another aspect that our approach considers is the individual uEV size; accordingly, besides determining total EVs, we could analyze and divide these structures into four subsets ranging from 0.1 to 0.9 μm (100 to 900 nm). Independently of the apoptotic bodies, the consensus indicates that EVs can be broadly classified into small and large extracellular vesicles. The small subset, typically referred to as exosomes, exhibits a diameter of 30–150 nm and originates from the endocytic pathway, while the large ones (microvesicles) have a mean diameter of 200–1000 nm and bud directly off the plasma membrane of the originating cells [3].

Although our results indicate that the most represented uEVs are the 0.1 μm-sized, the larger, less abundant 0.3 and 0.5 μm-sized subsets exhibited a more robust significance differentiating LN. This fact is supported by the results given by the constructed ROC curves, which confirmed that the 0.3 and 0.5 μm uEV concentrations constitute the best-analyzed parameters to differentiate the LN outcome, exhibiting significant and robust AUCs. Remarkably, the concentration of these larger microvesicles better correlates with renal-function-associated variables such as eGFR or creatinine, besides classical SLE descriptors such as SLEDAI, complement, or autoantibodies levels. Despite displaying similar correlation patterns, the largest analyzed uEVs of 0.9 mm are so scarce that their determination would become technically complex compared with the rest of the subsets. 

While we do not generate any data that could explain the differential concentration and size changes of uEVs during LN, it is interesting that all of them could be related to the inflammatory status of damaged kidneys. Accordingly, in different cell or organ contexts, it has been reported that inflammation induces altered EV biogenesis and release, including an increased size distribution of microglial cells upon lipopolysaccharide-induced activation [34] or during the progression of prostate cancer, where increased plasmatic exosome levels were detected [35]. 

Interestingly, some inflammatory mediators have been directly related to the cellular endocytic pathways controlling EV secretion. One of them is IL-13, a cytokine that has been found in high levels in active LN patients [36], and is known to positively regulate the expression of ALG-2-interacting protein X (ALIX) [37], which is a multifunctional player involved in multivesicular body biogenesis, endocytic membrane trafficking, and apoptosis [38]. Some studies have also documented that the overexpression of ALIX or its interactor, the tumor susceptibility gene 101 (TSG101), which is part of the endosomal sorting complex required for transport I (ESCRT-I), directly affects the size or the concentration of secreted EVs by HEK293 cells [39]. Accordingly, the inflammatory microenvironment in LN could favor the morphological and numerical changes in uEVs through the direct modulation of the endocytic pathways. This fascinating idea could be further analyzed in dedicated and independent studies.

Finally, and as mentioned before, uEVs constitute promising candidates for the discovery of noninvasive biomarkers to predict and monitor the evolution of kidney pathologies. Beyond their usefulness as stand-alone parameters, these vesicles could be incorporated into multiplex diagnostic platforms that overcome the limited sensitivity and specificity of classical kidney-damage-related biomarkers. Changes in EVs might occur even before there are variations in traditional blood-measured indicators of kidney function, such as creatinine and urea concentrations, thus allowing early diagnostic approaches. In this way, we propose that the concentration analysis of mid-size microvesicles (0.3–0.5 mm) in urine samples, followed by the concentration of total uEVs, could represent a useful LN biomarker. However, standardizing and validating our results in more extensive and prospective cohorts to gain insight into the value of these proposed parameters, even during the monitoring of nephritis response in patients upon treatment, is contingent on obtaining a better understanding of their value.

## Figures and Tables

**Table 1:** Main demographics, clinical and laboratory features of SLE/LN patients.

Variable	SLE, Median (IQR) *n* = 11	SLE with LN, Median (IQR) *n* = 15	*p*-Value
Age	28 (21–37)	27 (20–34)	0.3898
Female	10/11	8/15	0.2691
SLEDAI	4 (4–8)	20 (16–22)	**0.0001**
White blood cell count	4.45 (4.13–5.00)	7.10 (3.55–9.98)	0.2141
Creatinine (serum)	0.72 (0.62–0.81)	2.47 (1.50–3.99)	**<0.0001**
eGFR	103.50 (115.10–121.20)	28.70 (15.80–49.30)	**<0.0001**
Creatinine (random urine)	51.00 (37.00–127.00)	76.00 (44.00–171.80)	0.2753
C3	97.00 (71.00–114.00)	48.50 (37.75–58.75)	**0.0001**
C4	15 (11–21)	8 (8–13)	0.0663
Anti-dsDNA	35.80 (10.38–117.00)	425.00 (33.48–746.90)	**0.0428**
Classification of Lupus Nephritis by ISN/RPS (*n*)			
Class IV	-	2	-
Class III + V	-	2	
Class IV + V	-	11	

The differences among groups were analyzed with Mann–Whitney tor χ^2^ tests. Statistically significant values are shown in bold text. IQR—interquartile range; SLEDAI—Systemic Lupus Erythematosus Disease Activity Index; eGFR—estimated Glomerular Filtration Rate; C3—complement component 3; C4—complement component C4; anti-dsDNA—antibodies anti-double stranded DNA.

**Table 2:** EV size-subset concentrations in SLE/LN patients.

EV Size-Subset	SLE [EVs/μL], Median (IQR)	SLE with LN [EVs/μL], Median (IQR)	*p*-Value
0.1 μm	28.84 (3.59–43.70)	112.40 (36.34–1573.00)	**0.0128**
0.3 μm	0.80 (0.10–1.46)	7.41 (1.07–64.86)	**0.0019**
0.5 μm	0.38 (0.05–0.57)	1.44 (0.43–16.21)	**0.0031**
0.9 μm	0.24 (0.09–0.46)	0.72 (0.16–9.28)	0.0589

The differences among groups were analyzed with Mann–Whitney U test. Statistically significant values are shown in bold text.

## Data Availability

The data that support the findings of this study are available from the corresponding authors upon reasonable request.
